# Probability of Detection and Defect Distribution Modeling of Porous Hard-Alpha Inclusions in Titanium Aero-Engine Disks

**DOI:** 10.3390/ma19050911

**Published:** 2026-02-27

**Authors:** Hongzhuo Liu, Puying Shi, Zhengli Hua, Dawei Huang, Xiaojun Yan

**Affiliations:** 1 School of Energy and Power Engineering, Beihang University, Beijing 100191, China; liuhongzhuo@buaa.edu.cn (H.L.);; 2 Western Superconducting Technologies Co., Ltd., Xi’an 710018, China; 3 China National Key Laboratory of Science and Technology on Aero-Engine Aero-Thermodynamics, Beijing 100191, China; 4 Beijing Key Laboratory of Aero-Engine Structure and Strength, Beijing 100191, China; 5 Collaborative Innovation Center of Advanced Aero-Engine, Beijing 100191, China

**Keywords:** ultrasonic testing (UT), porous hard-alpha inclusions, non-destructive evaluation, probability of detection (POD), defect distribution, aero-engine disk, probabilistic damage tolerance

## Abstract

A major quality challenge in the application of titanium alloys is the persistence of substances known as “hard-alpha inclusions”. Although hard-alpha inclusions are extremely rare and typically small in size in high-quality titanium alloys for aero-engine disks, their hard and brittle nature poses a non-negligible threat to the structural integrity of the disks. Due to the extreme scarcity of natural hard-alpha inclusions, most previous studies have focused on “synthetic dense hard-alpha particles” rather than “real porous hard-alpha inclusions”, inevitably over-looking the differences between them. In this work, a method of introducing titanium nitride sponge preforms into the electrode preparation step of the smelting process is proposed and implemented, successfully fabricating real porous hard-alpha inclusions in TC4 titanium alloy disks. On this basis, the detection characteristics of ultrasonic non-destructive testing for such porous hard-alpha inclusions are investigated, and a probability of detection (POD) model for these defects is established for the first time. A defect distribution model of porous hard-alpha inclusions for the probabilistic damage tolerance assessment of disks is also derived. This work reveals that, unlike the “linear” behavior of traditional models, the new defect distribution model adheres to a “cubic polynomial” relationship.

## 1. Introduction

Titanium alloys exhibit prominent advantages, including high specific strength, superior corrosion resistance, a wide operating temperature range and excellent fatigue performance, thus being extensively adopted for fabricating critical aero-engine components such as fan disks and compressor disks [1,2,3,4]. Despite the implementation of stringent process controls in the production of high-quality titanium alloys for aerospace applications, existing manufacturing processes still cannot completely eliminate a typical metallurgical defect known as hard-alpha inclusions. These defects are primarily composed of titanium–nitrogen compounds, characterized by high melting points and high hardness and brittleness, and are highly prone to acting as fatigue crack initiation sites under cyclic loading [5,6,7]. According to statistics from the U.S. Federal Aviation Administration (FAA), as many as 76% of previous aviation accidents caused by metallurgical defects in titanium alloys were associated with hard-alpha inclusions (19 out of 25 accidents recorded between 1962 and 1990) [8]. A case in point is the Sioux City crash that occurred on 19 July 1989. The CF6-6 engine mounted on the DC-10 aircraft involved in the accident suffered an uncontained fan burst failure, which resulted in 111 fatalities [9]. This accident was precisely caused by the propagation of fatigue cracks initiated by hard-alpha inclusions near the core of the fan disk under low-cycle loading conditions [10].

To address the structural reliability issues of aero-engines caused by hard-alpha inclusions in titanium alloys, researchers have developed two technical approaches. On one hand, efforts are devoted to investigating the smelting process of titanium alloys and the dissolution behavior of hard-alpha inclusions, aiming to maximize the elimination of such defects during production [11,12]. On the other hand, the possibility of the presence of hard-alpha inclusions under current manufacturing processes is accepted, and the probabilistic damage tolerance theory is adopted to mitigate potential risks. This approach treats hard-alpha inclusions as initial cracks and employs probabilistic fracture mechanics to calculate failure risks, with the probability of detection (POD) model and defect distribution model serving as the key input conditions for its implementation [13]. The proposal of this approach prompted the U.S. Federal Aviation Administration (FAA) to issue airworthiness regulations AC 33.70-1 and AC 33.70-3, which specify detailed protocols for quantitatively assessing failure risks induced by hard-alpha inclusions using the aforementioned two models and stipulate that only compliant products can obtain airworthiness certification [14,15]. Similarly, the European Union Aviation Safety Agency (EASA) and the Civil Aviation Administration of China (CAAC) have also issued relevant airworthiness regulations with the same requirements [16,17]. Today, the probabilistic damage tolerance theory based on the POD model and defect distribution model has become a crucial approach to resolving structural integrity issues induced by defects [18,19,20,21,22].

Due to the extremely rare occurrence of hard-alpha inclusions in field data, conducting comprehensive research on such defects remains challenging. To address this limitation, the use of synthetic hard-alpha (SHA) as a substitute has been proposed by researchers, which effectively mitigates the issue of inadequate real-world data. A technical route for fabricating SHA using the hot isostatic pressing (HIP) process was initially established, and the equivalence of the fatigue performance of SHA to that of naturally occurring hard-alpha inclusions was demonstrated [23]. Building upon this finding, the FAA coordinated a collaborative effort involving five organizations—General Electric Aviation, Rolls-Royce, Pratt & Whitney, and two others—to develop a POD model for SHA in synthetic inclusion disks [24,25,26,27]. Subsequently, other studies were conducted in which SHA inclusions were implanted into titanium alloy bars, followed by ultrasonic testing (UT) to establish a defect distribution model for SHA [28,29].

It should be noted that recent studies have confirmed the absence of any observable porosities in SHA [30], a characteristic that is markedly distinct from naturally occurring hard-alpha inclusions. Given that the presence of porosities is a critical factor governing UT responses [31,32], it can be reasonably inferred that their non-destructive testing (NDT) characteristics may differ. In fact, SHA is fabricated using a specific HIP process, thus typically exhibiting a dense microstructure. In contrast, naturally occurring hard-alpha inclusions are most likely induced by titanium nitride sponge, which is inherently porous [33]. Although molten metal may infiltrate some of these pores during the smelting process, the pores within naturally occurring hard-alpha inclusions are unlikely to be completely eliminated in the final titanium alloy product. Studies have shown that their residual porosity can range from approximately 1% to 23% [6]. In addition, unlike the well-defined cylindrical geometry and uniform dimensions of SHA, porous hard-alpha inclusions exhibit complex geometries and random sizes, leading to distinct ultrasonic response characteristics for these two types of defects. Given these inherent differences, it is essential to conduct dedicated research on the modeling of the POD and defect distribution specifically for porous hard-alpha inclusions.

This work presents a technical methodology involving the incorporation of titanium nitride sponge embedded bodies during the electrode preparation stage prior to smelting. Using this approach, titanium alloy disks containing porous hard-alpha inclusions are fabricated. Subsequently, NDT is conducted to analyze the ultrasonic response. Industrial computed tomography (CT) inspections are then performed to reconstruct the three-dimensional geometric morphology of the defects and determine their precise dimensional characteristics. Finally, both the POD model and defect distribution model are established for the porous hard-alpha inclusions in titanium alloy disks.

## 2. Materials and Methods

This section outlines the preparation of defective disks and associated NDT procedures. First, pre-embedded particles of porous titanium nitride sponge are fabricated. Subsequently, these particles are incorporated into the titanium sponge material for TC4 alloy production, yielding bars with hard-alpha inclusions. The bars are then forged into disks. Next, UT is performed on the defective disks and sampled bars, and high-energy CT scans are conducted on subsets of these sampled bars. Finally, representative hard-alpha inclusions are selected for sectioning, followed by composition and metallographic analyses.

### 2.1. Porous Titanium Nitride Sponge

**Figure 1:**
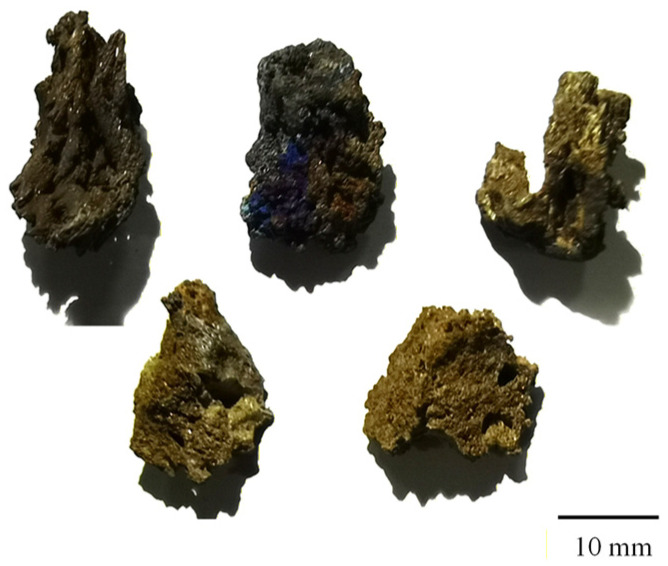
Geometric morphology of titanium nitride sponge.

Studies have shown that up to 26 potential inducements exist for the formation of hard-alpha inclusions, with titanium nitride (TiN) and titanium oxide (TiO_2_) mixed in titanium sponge identified as the most prominent ones [33,34]. Despite the dedicated efforts to eliminate TiN from titanium alloys by virtue of continuously optimized smelting processes and simulation technologies, its complete removal has remained unachievable to date [35,36,37]. The core process for titanium sponge production via the Kroll method is composed of three key steps: chlorination purification, magnesiothermic reduction, and distillation separation. Industrially, an integrated reduction–distillation process is widely adopted, where reduction and distillation are sequentially conducted in the same sealed reactor. Throughout the entire process, argon is utilized as the inert protective gas to isolate air from the reaction system [38]. During the magnesiothermic reduction stage, if the reactor seal is compromised, air will infiltrate the reaction system, thus triggering rapid oxidation and nitridation of titanium sponge and reaction intermediates (lower-valent titanium chlorides) when they react with O_2_ and N_2_ in the air [11,39]. To simulate this defect formation mechanism, the original sealed argon protective atmosphere of the reactor is modified, and slight leakage is artificially controlled to allow quantitative air infiltration and participation in the reaction. Ultimately, 30 specimens of titanium nitride sponge are successfully prepared. Detection results demonstrate that these embedded particles possess a nitrogen content of 10–15% and a maximum cross-sectional diameter of 5–20 mm, and they are characterized by a porous structure and irregular morphology (Figure 1).

### 2.2. Disks Containing Hard-Alpha Inclusions

The melting point of monolithic TiN is 3290 °C, markedly higher than the typical temperature of the vacuum arc remelting (VAR) molten pool for titanium alloys [6,40]. Research has documented that the dissolution rate of monolithic TiN in diverse titanium alloy melts spans 0.37–30 μm/s [41], and the dissolution kinetics of monolithic TiN is predominantly governed by nitrogen mass transfer and strongly dependent on the molten pool temperature and fluid velocity [42]. By contrast, the dissolution behavior of porous titanium nitride sponge is more intricate and is significantly affected by temperature [43]. Consequently, precise regulation of melting parameters is indispensable for fabricating samples containing porous hard-alpha inclusions from titanium nitride sponge.

**Figure 2:**
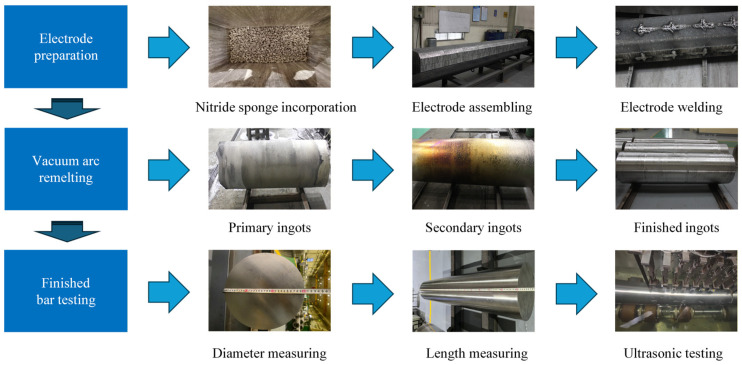
The preparation process of bars containing hard-alpha inclusions.

During the titanium alloy electrode preparation stage, the titanium nitride sponge fabricated in Section 2.1 is mixed into 3600 kg of TC4 sponge titanium and then compacted into electrodes at a predetermined pressure of 25 MPa using a hydraulic press. After electrode pressing, a consumable electrode is obtained by welding in a vacuum plasma welding chamber, followed by ingot preparation through three passes of VAR. During the VAR process, the maximum melting current is controlled so that it does not exceed 29 kA, and the maximum melting rate is limited to 24 kg/min. The feeding stage is initiated when 220 kg of the consumable electrode remains. After completion of ingot melting, the power is tripped, and the ingot is cooled for 6 h before being discharged from the furnace. Subsequent to ingot preparation, forging and surface machining are performed to produce two bars with specifications of Φ300 × 2000 mm. Following the acquisition of the bars, their dimensions are measured, and multi-zone water immersion ultrasonic testing is conducted to detect potential defects. The preparation process of the bars containing hard-alpha inclusions is illustrated in Figure 2.

**Figure 3:**
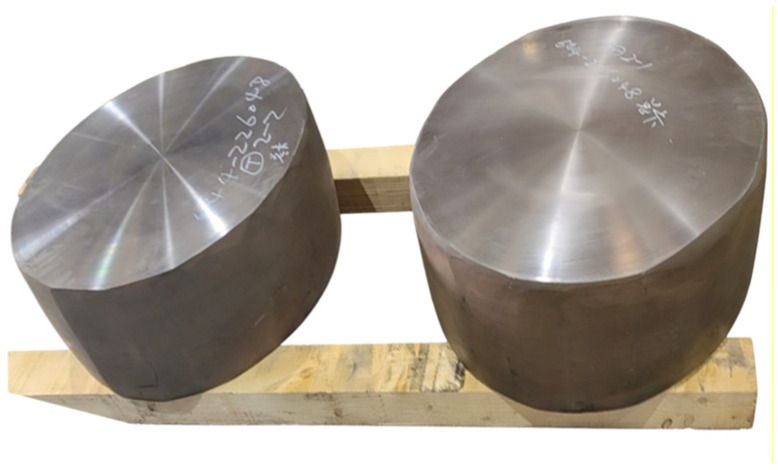
Disks containing hard-alpha inclusions.

Regions with a relatively high number of defects detected by ultrasonic testing are cut from the two bars, each with dimensions of Φ300 × 1150 mm. These regions are then subjected to forging processing. A single heating cycle is adopted, with upsetting performed in 3–4 passes and a deformation of 20–30% per pass; the heating temperature is set at 970 °C with a holding time of 210 min. Air cooling is applied after the completion of upsetting. Finally, two disks with specifications of Φ625 × 310 mm are obtained, as illustrated in Figure 3.

### 2.3. UT Experiments on Defective Specimens

Immersion ultrasonic testing is performed on defective TC4 disks. To facilitate the experiment, two disks with dimensions of Φ625 × 300 mm are cut into four disks of Φ625 × 150 mm using low-speed wire electrical discharge machining (WEDM). The ultrasonic testing experiment employs KT-A1500 (Waygate Technologies, Hürth, Germany) equipment, equipped with a 10Z10SJT-type (China United Test & Certification Co., Ltd., Beijing, China) probe and operating at a detection frequency of 10 MHz [44]. A series of TC4 calibration blocks with Φ0.8 mm flat-bottom holes (FBHs) are adopted. To investigate the influence of detection sensitivity on the test results, two detection sensitivity conditions are set based on Φ0.8 mm FBHs and Φ1.2 mm FBHs, respectively. During detection, defects are identified using real-time C-scan images. After detection completion, inspectors evaluate the A-scan signals of detected defects and record their equivalent sizes and position coordinates.

**Figure 4:**
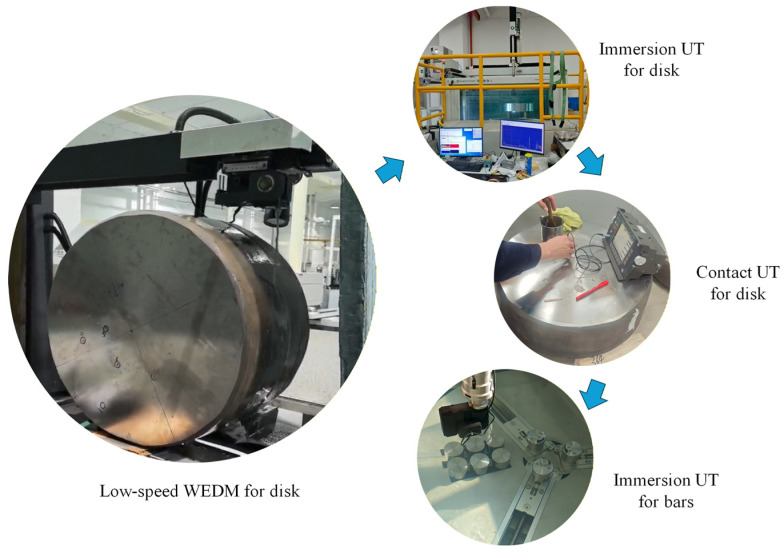
WEDM and UT experiments for specimens containing hard-alpha inclusions.

Based on the information obtained from the immersion ultrasonic testing of the disks, contact ultrasonic testing is adopted to reconfirm the defect positions within the disks, with corresponding markings made on the disk surfaces. Subsequently, cylindrical blocks containing defects are extracted from the disk at the marked locations via low-speed WEDM, resulting in a total of 21 bars with dimensions of Φ60 × 150 mm. Immersion ultrasonic testing is performed on these 21 bars using the LS-200LP-1200 (ScanMaster Systems (IRT) Ltd., Kfar Saba, Israel) system equipped with an IX0519GB (TLC Ultrasound, New Milford, CT, USA) probe operating at 5 MHz [45]. The tests are also conducted under two detection sensitivity conditions based on Φ0.8 mm FBHs and Φ1.2 mm FBHs, respectively. The WEDM cutting of disks and ultrasonic testing experiments are illustrated in Figure 4.

### 2.4. High-Energy CT Size Measurement

**Figure 5:**
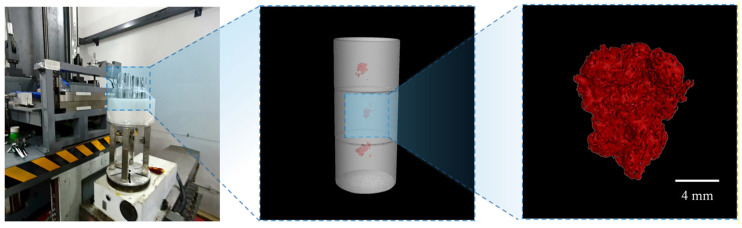
3D morphology of defects characterized by high-energy CT.

High-energy CT scanning is conducted on 10 out of the 21 bars (refer to UT experiments in Section 2.3) to enable precise measurement of defect dimensions [46]. The tests are performed using a 6 MeV linear accelerator CT platform (Beijing Granpect Technology Co., Ltd., Beijing, China) [47]. A third-generation CT cone-beam scanning mode is adopted, with a focal spot size of 0.5 mm, scanning height of 200 mm, scanning angle of 720°, pulse number per frame of 180, sampling number per rotation of 720, and interlayer interval of 0.1 mm. Subsequently, the 3D geometric morphologies of hard-alpha inclusions are reconstructed (Figure 5), as detailed in Appendix A.

### 2.5. Composition and Metallographic Analysis

**Figure 6:**
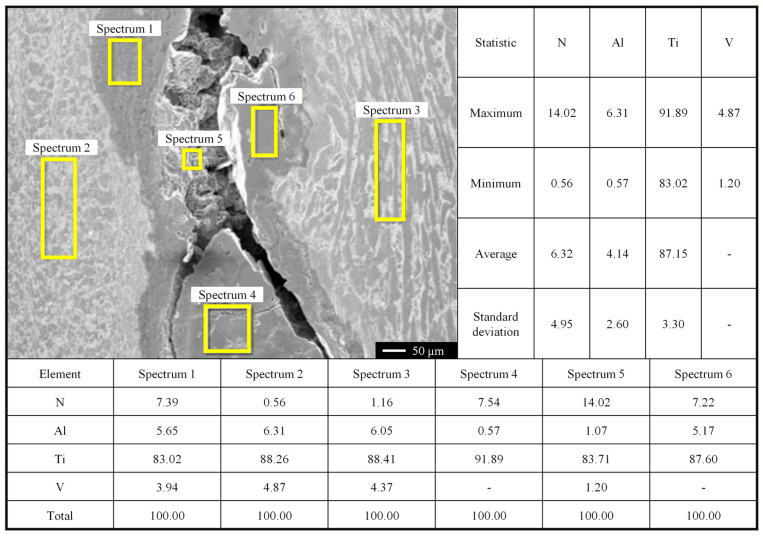
Chemical composition analysis of a representative hard-alpha inclusion.

Chemical composition characterization is performed on hard-alpha inclusions in sampling specimens. A typical hard-alpha inclusion is selected for EDS analysis to determine the composition of the inclusion. As shown in Figure 6, this inclusion conforms to the characteristics of naturally occurring hard-alpha inclusions, with an average nitrogen content of 6.32% in the TiN region.

**Figure 7:**
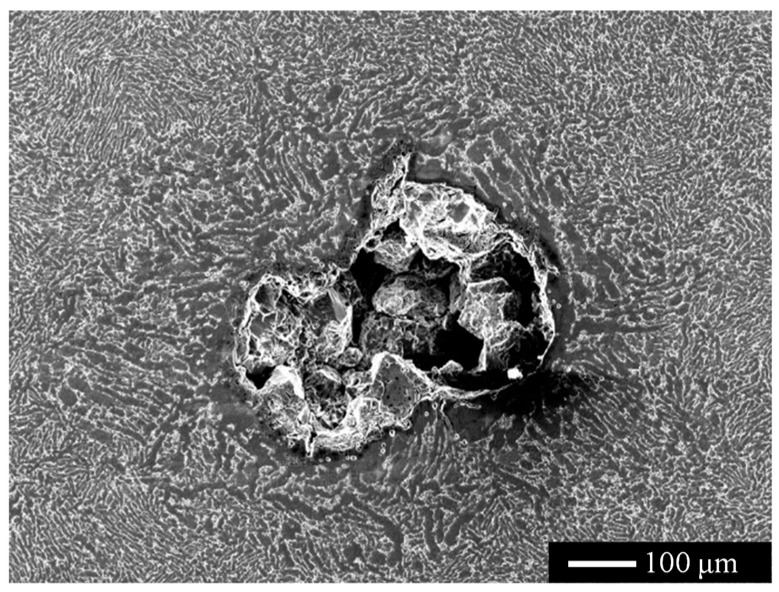
SEM morphology of a representative hard-alpha inclusion.

A representative hard-alpha inclusion is selected to examine the characteristic morphologies of its core region and diffusion zone. As illustrated in Figure 7, SEM images reveal that the hard-alpha inclusion exhibits clearly distinguishable core and diffusion zones. Metallographic cross-sectional analysis indicates that the core region contains a hollow pore structure. It can therefore be inferred that the core region possesses a porous structure prior to metallographic preparation. During the grinding process, certain discontinuous brittle phases are removed, which further enlarges the pores and results in an almost continuous pore network, as observed in the figure.

## 3. Results and Discussion

In this section, the POD model and the defect distribution model are derived based on the experimental results. First, the POD model for hard-alpha inclusions is established using the detection data obtained from UT of the disks and 21 sampled bars as well as CT scans of 10 sampled bars. Then, the exceedance number of the defect distribution of hard-alpha inclusions is deduced from the POD model. Subsequently, the baseline defect distribution model is obtained by fitting the exceedance number. Next, the post-inspection defect distribution model is derived through the combination of the POD model and the baseline defect distribution model. Finally, a comparison is conducted with the requirements specified in AC 33.70-3.

### 3.1. NDT Data Processing and POD Modeling

To establish the defect distribution model, it is essential to first develop the POD model. Various approaches have been proposed for POD modeling. Some researchers have employed classical statistical methods, approximating detection probability through frequency based on extensive repeated inspection experiments [28]. However, more widely accepted approaches are the two methods based on specific statistical models recommended in the U.S. military standard MIL-HDBK-1823A: the “hit/miss” method and the “*â* vs. *a*” method. The “hit/miss” method uses the actual defect size and the binary detection outcome (detected or not detected, coded as 1 or 0) as input parameters, whereas the “*â* vs. *a*” method utilizes the actual defect size and the corresponding defect response signal as inputs [48].

The “*â* vs. *a*” method is adopted for POD modeling in this work, and the definitions of the defect response signal *â* and the actual defect size *a* used in this study are specified as follows.

**Figure 8:**
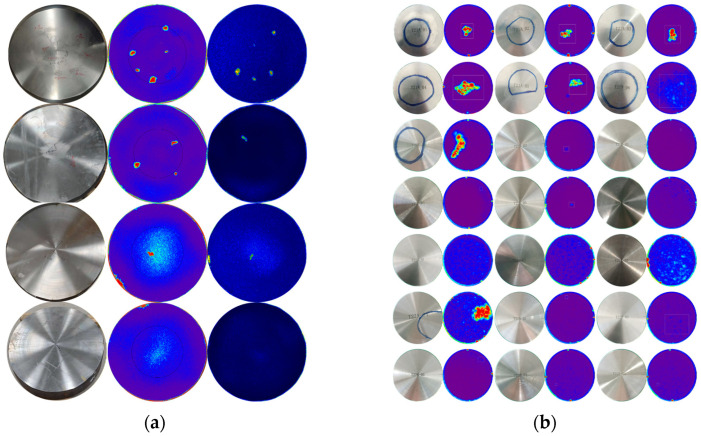
Specimens and their ultrasonic C-scan images: (**a**) Φ625 × 150 mm disks; (**b**) Φ60 × 150 mm bars.

Defect response signal *â*: The immersion ultrasonic C-scan images corresponding to the disks and sampled bars are presented in Figure 8. For each ultrasonic C-scan image of a defect, regions with prominent defect response signals are identified, and the maximum value of the A-scan signal intensity in these regions is extracted. This maximum value is used to calculate the equivalent flat-bottom hole (EFBH) diameter of the defect. The equivalent flat-bottom hole area corresponding to the EFBH is defined as the defect response signal â used for POD model establishment. That is,

(1) $$\hat{a}=a_{\mathrm{EFBH}}=\frac{S}{S_{C}}\frac{\pi D_{C}^{2}}{4}$$

where *S* denotes the A-scan signal intensity of the defect (in units of % full-screen height (FSH)), *S*_C_ represents the A-scan signal intensity of the calibration flat-bottom hole (in units of % FSH), and *D*_C_ indicates the diameter of the calibration flat-bottom hole.

**Figure 9:**
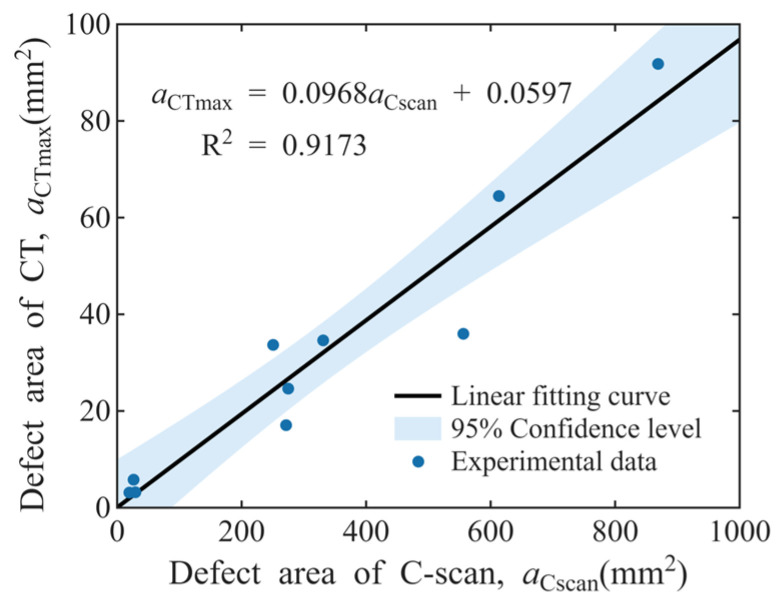
Linear regression between $a_{\mathrm{Cscan}}$ and $a_{\mathrm{CT}\max}$.

Actual defect size *a*: The area of the maximum cross-section of defects perpendicular to the ultrasonic beam $a_{\mathrm{CT}\max}$ is extracted from the 3D defect models obtained via high-energy industrial CT scanning, and this area is defined as the actual defect size *a*. For the 21 sampled bars involved in this work, $a_{\mathrm{CT}\max}$ for 10 samples is obtained through direct measurement via CT tests. For the remaining 11 samples, $a_{\mathrm{CT}\max}$ is derived from linear fitting of the defect area in ultrasonic C-scan images $a_{\mathrm{Cscan}}$. Here, it is assumed that there exists a simple linear relationship between $a_{\mathrm{Cscan}}$ and $a_{\mathrm{CT}\max}$:

(2) $$a_{\mathrm{CT}\max}=p\cdot a_{\mathrm{Cscan}}+q$$

where *p* and *q* are the slope and intercept of the linear equation, respectively. Shown in Figure 9 are the linear regression results of 10 bar samples with known $a_{\mathrm{CT}\max}$ and $a_{\mathrm{Cscan}}$. It can be seen that the experimental data are basically within the 95% confidence interval of the regression line, and the value of *R*^2^ = 0.9173 indicates a good linear relationship between the two sets of data. By substituting the $a_{\mathrm{Cscan}}$ values of the remaining 11 defects into Equation (2), the estimated values of the $a_{\mathrm{CT}\max}$ can be obtained.

Thus, the defect response signal *â* and the actual defect size *a* are clearly defined. By applying the results corresponding to the UT experiments in Section 2.3 to this definition, valid input conditions for POD modeling are obtain ed, as detailed in Appendix B. Typically, there are four possible linear relationships between *â* and *a*, namely *â* vs. *a*, log(*â*) vs. *a*, *â* vs. log(*a*), and log(*â*) vs. log(*a*) [48]. Through regression analysis, the log(*â*) vs. log(*a*) model with the optimal fitting degree is selected, which means that the following log-linear relationship exists between the defect response signal *â* and the actual defect size *a*:

(3) $$\log(\hat{a})=\beta_{0}+\beta_{1}\log(a)+\varepsilon ,\;\varepsilon ∼N(0,\tau^{2})$$

where *β*_0_, *β*_1_ and *ε* are model parameters, corresponding to the intercept, slope, and error of the log-linear regression, respectively. The POD(*a*) is a function of the NDT decision threshold *â*_dec_, linear equation regression coefficients *β*_0_ and *β*_1_, and error variance *τ*^2^, as expressed below:

(4) $$\begin{array}{ll} POD(a) & =Probability[\hat{a}>{\hat{a}}_{dec}]=Probability[log(\hat{a})>\log({\hat{a}}_{dec})]\\ & =1-\Phi [\frac{\log({\hat{a}}_{dec})-[\beta_{0}+\beta_{1}\log(a)]}{\tau }]\\ & =\Phi [\frac{\log(a)-[\log({\hat{a}}_{dec})-\beta_{0}]/\beta_{1}}{\tau /\beta_{1}}]\\ & =\Phi [\frac{\log(a)-\mu }{\sigma }] \end{array}$$

where *μ* and *σ* represent the mean and variance of the lognormal distribution accumulation function respectively, with their expressions defined as follows:

(5) $$\left\{\begin{array}{l} \mu =\frac{\log({\hat{a}}_{dec})-\beta_{0}}{\beta_{1}}\\ \sigma =\frac{\tau }{\beta_{1}} \end{array}\right.$$

**Figure 10:**
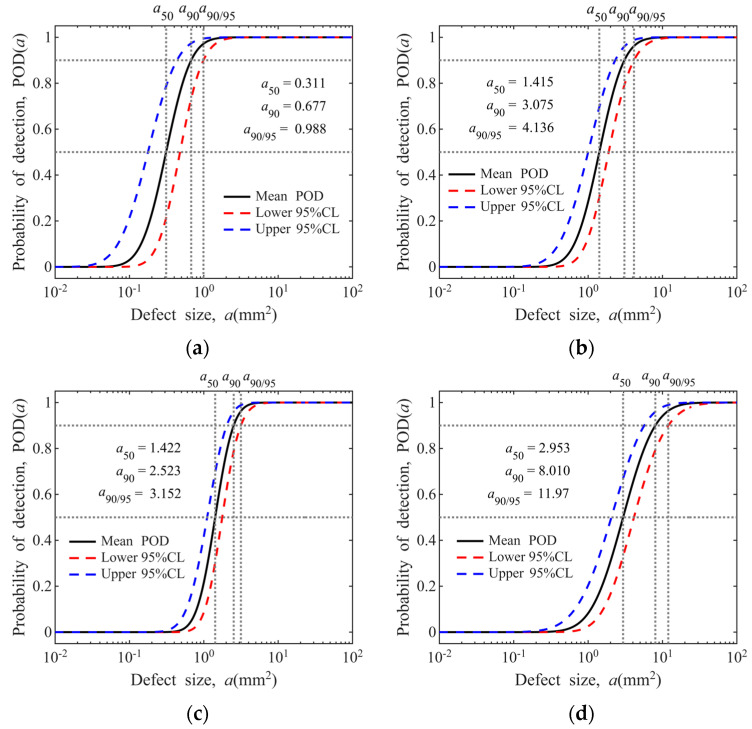
POD model for hard-alpha inclusions: (**a**) sensitivity for Φ0.8 mm FBH in disks; (**b**) sensitivity for Φ1.2 mm FBH in disks; (**c**) sensitivity for Φ0.8 mm FBH in bars; (**d**) sensitivity for Φ1.2 mm FBH in bars.

The POD model for hard-alpha inclusions is presented in Figure 10. As illustrated in the figure, detection sensitivity exerts a significant influence on the POD. In addition, the POD varies considerably with different specimen types, which may be attributed to discrepancies in detection frequency and specimen dimensions [48,49].

### 3.2. Deriving Defect Distribution from POD Model

A method integrating UT experimental data and POD model derivation is adopted to establish the defect distribution model [13,28]. Drawing on established methodologies, a simplified hypothesis correlating the POD model with defect counts is proposed to deduce the defect distribution model, and its mathematical expression is given as follows [50]:

(6) $$f=\frac{X}{POD}$$

where *X* is the number of defects detected through field NDT and *f* represents the estimated total (both detected and undetected) number of defects.

In fact, the transformation of Equation (6) into X=f⋅POD is independent of defect types and the NDT methods adopted. The number of defects *X* detected in field applications is determined by the product of the POD and the true number of defects $f_{\mathrm{true}}$, which is a self-evident fundamental relationship. However, the limitation of this method is that the true number of defects $f_{\mathrm{true}}$ in a material is an intrinsic physical characteristic of the material itself. When Equation (6) is applied in its fractional form, what we obtain is an estimated value $f_{\mathrm{estimated}}$ of the true number of defects. That is, different estimates of the defect number can be derived from the selection of different field data *X* values and different POD models.

From this analysis, the estimated total number of defects of a given size is derived. Additionally, the cumulative number of defects exceeding the specified size can be calculated, enabling the establishment of the defect exceedance number model [50]:

(7) $$F_{exc}(a)=\sum_{a}^{a_{sup}}\frac{X(a_{i})}{POD(a_{i})}$$

where *a* represents a specific defect size, *a*_sup_ is the supremum limit of detectable defect size, POD(*a*) is the probability of the detection function of a defect, and *X*(*a*) is the distribution function of the defects that have already been detected, which is assumed to be a uniform distribution in the absence of field data [13,50]. The impact of distributional assumptions of field data is presented in Appendix C.

**Figure 11:**
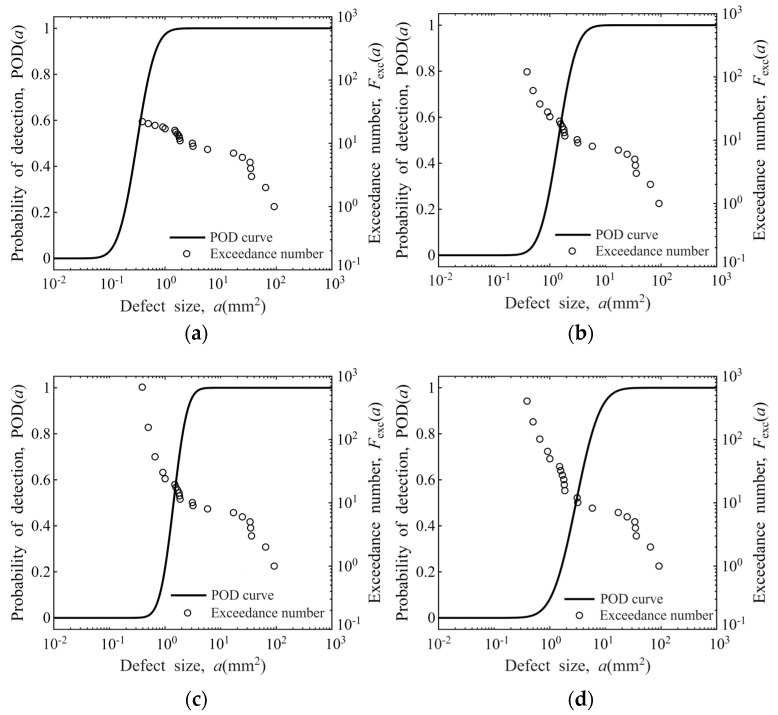
Derivation of defect distribution exceedance numbers from the POD model: (**a**) sensitivity for Φ0.8 mm FBH in disks; (**b**) sensitivity for Φ1.2 mm FBH in disks; (**c**) sensitivity for Φ0.8 mm FBH in bars; (**d**) sensitivity for Φ1.2 mm FBH in bars.

The estimated exceedance numbers derived from the proposed methodology are presented in Figure 11. As demonstrated in the figure, the exceedance number calculations are significantly affected by the steep-slope region of the POD curve, during which minor variations in defect size correspond to substantial changes in probability of detection.

### 3.3. Defect Distribution Model for Hard-Alpha Inclusions

As the defect distribution has been derived by the POD model in Section 3.2, analytical expressions are adopted to establish the defect distribution model in this section. The classical log-log linear model is first considered, and its expression is given as follows [11]:

(8) $$\log(F_{exc}(a))=m\cdot \log(a)+n$$

where *m* and *n* denote the slope and intercept of the regression line in the log-log coordinate system, respectively.

**Figure 12:**
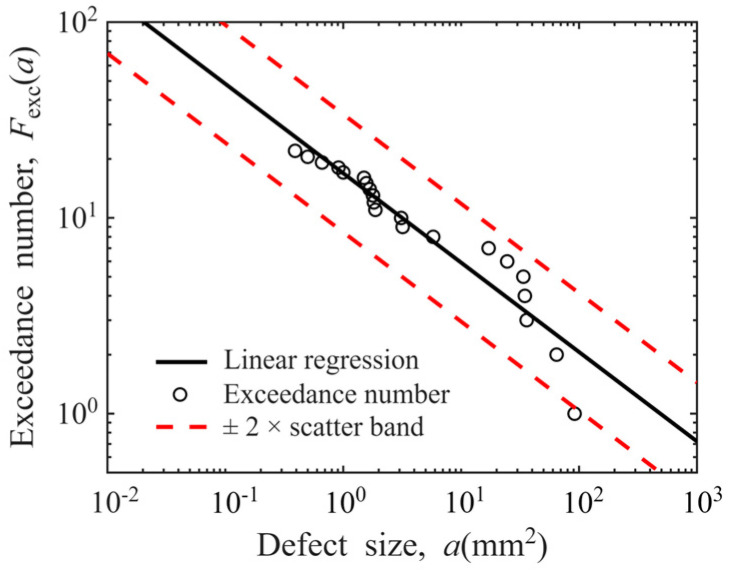
Defect distribution model of hard-alpha inclusions (Φ0.8 mm FBH sensitivity in disks).

Taking the data of Φ0.8 mm FBH sensitivity in disks as an example, its log-log linear defect distribution model is presented in Figure 12. The ordinates of “○” represent the estimated exceedance numbers of defects, and their abscissa correspond to the actual sizes of 21 defects. A fitting line in the log-log coordinate system (*R*^2^ = 0.9043) is included. Most estimated exceedance numbers are observed to fall within the ±2× scatter band, confirming that the log-log linear relationship adequately characterizes the POD-derived defect distribution with the sensitivity of Φ0.8 mm FBH in disks.

**Figure 13:**
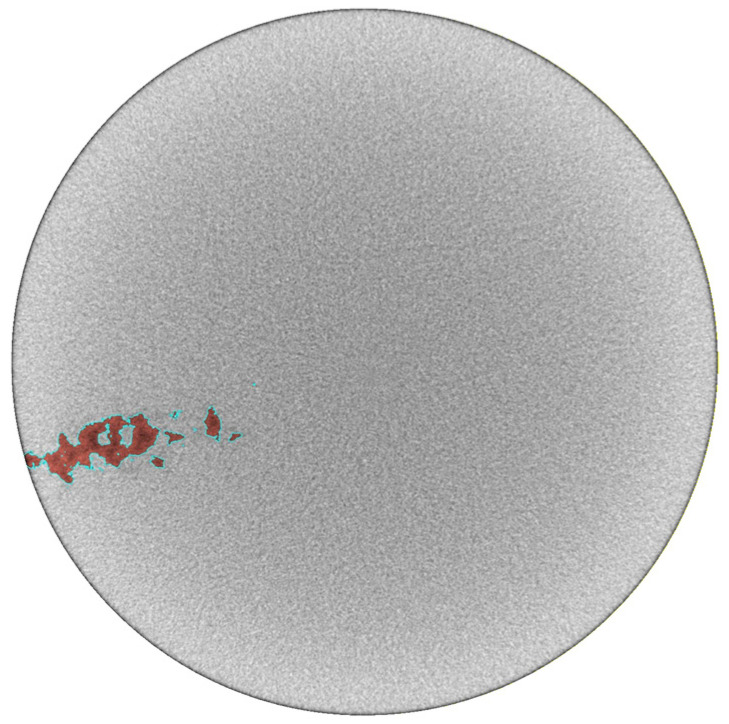
2D slice of the maximum cross-sectional area $a_{\mathrm{CT}\max}$ of the outlier defect.

For the single outlier in Figure 12 that falls outside the ±2× scatter band, we analyzed the cause of this deviation using 2D slices from CT scanning. As shown in Figure 13, this cross-section corresponds to the location of the maximum area of the associated defect identified via CT scanning, where the area of the red region enclosed by the cyan outline is recorded as $a_{\mathrm{CT}\max}$. It can be observed that the defect cross-section is not fully connected and contains multiple incompletely filled regions. This issue stems from the grayscale segmentation algorithm used by the program for defect area identification, which classifies the unfilled internal regions of the defect as matrix material and thus underestimates the actual defect area to a certain extent.

**Figure 14:**
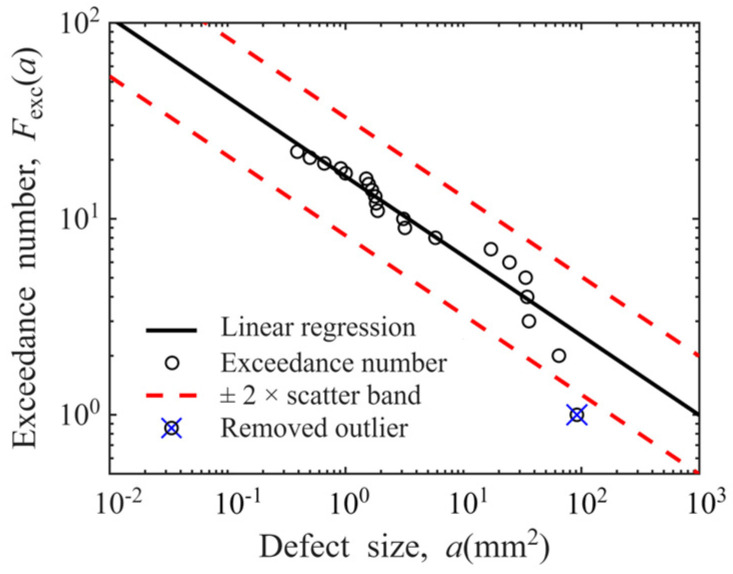
Defect distribution model after outlier removal (Φ0.8 mm FBH sensitivity in disks).

If this result is regarded as an outlier caused by random errors in experimental measurement and excluded from the linear regression process, the recalculated defect distribution curve is presented in Figure 14. The coefficient of determination *R*^2^ of the new fitted line is 0.9352, and all estimated exceedance numbers fall within the ±2× scatter band of the regression line. This change alters the slope of the regression line from the original −0.4566 to −0.4061, representing an 11.06% variation. This indicates that the data of large-sized defects has a significant impact on the shape of the defect distribution curve, and it is therefore necessary to discuss whether to retain this data point.

On the one hand, regarding this specific data point in the present experiment, it corresponds to a defect with a measured cross-sectional area of 91.81 mm^2^. Considering that in actual production processes, such a large defect would be sufficiently prominent to be detected during the ultrasonic testing stage of blank bars and thus rejected as a non-conforming product, eliminating any possibility of its presence in finished disks. From this perspective, excluding this outlier data point during defect distribution modeling is reasonable.

On the other hand, a comparison of Figure 12 and Figure 14 reveals that the regression line becomes significantly flatter after outliers are removed and recalculations are performed. This change is equivalent to increasing the number of larger-sized defects (which are more easily detectable) and decreasing the number of smaller-sized defects (which are harder to detect) in the defect distribution model. This yields an interesting result: removing data for a single large-sized defect actually leads to an increase in the number of large-sized defects predicted by the defect distribution model. As indicated by the analytical formula for calculating the probability of failure (POF) for disks presented in Equation (9), any variation in the probability distribution function $f_{D}\left(a_{0}\right)$ associated with the defect distribution model will impact the final calculated POF in the form of a product term within the integral equation [51]. Deliberately excluding an outlier data point may trigger unforeseen cascading effects on subsequent applications of the model. From this perspective, all data points should be retained with prudence during the defect distribution modeling process.

(9) $$\begin{array}{l} POF=V\cdot P_{d}\cdot ∭{∭}_{\Omega }f_{D}(a_{0})f_{s}(\sigma )f_{v}(\frac{da}{dN})f_{FT}(K_{IC})f_{NDI}(N)\\ \;\;f_{NDI}(POD)\;da_{0}\;d\sigma \;d(\frac{da}{dN})\;dK_{IC}\;dN\;d\;POD \end{array}$$

**Figure 15:**
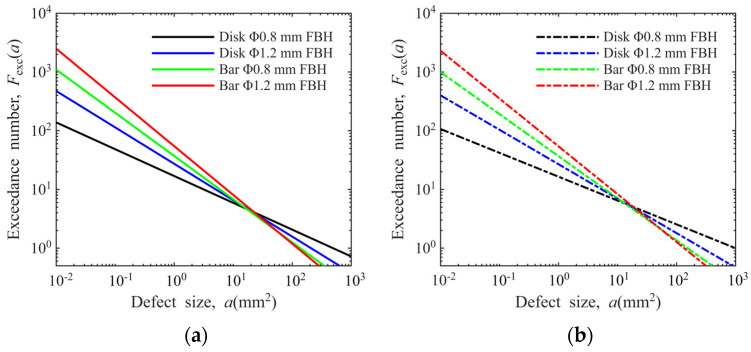
Linear regression model for defect distribution: (**a**) outliers retained; (**b**) outliers excluded.

Based on the above two reasons, we perform calculations with outliers retained and excluded, respectively. The defect distribution curves obtained under different conditions are shown in Figure 15. The slopes, intercepts, and *R*^2^ of the linear regression equations are presented in Table 1. As can be seen from the figure, experimental conditions have an impact on the slope of the defect distribution curve. An analysis of the *R*^2^ of each linear regression equation in Table 1 reveals that only 1/4 of the curves have an *R*^2^ exceeding 0.9, with the minimum *R*^2^ value being 0.7308. This indicates that the log-log linear relationship cannot effectively describe the defect distribution characteristics under all conditions.

**Figure 16:**
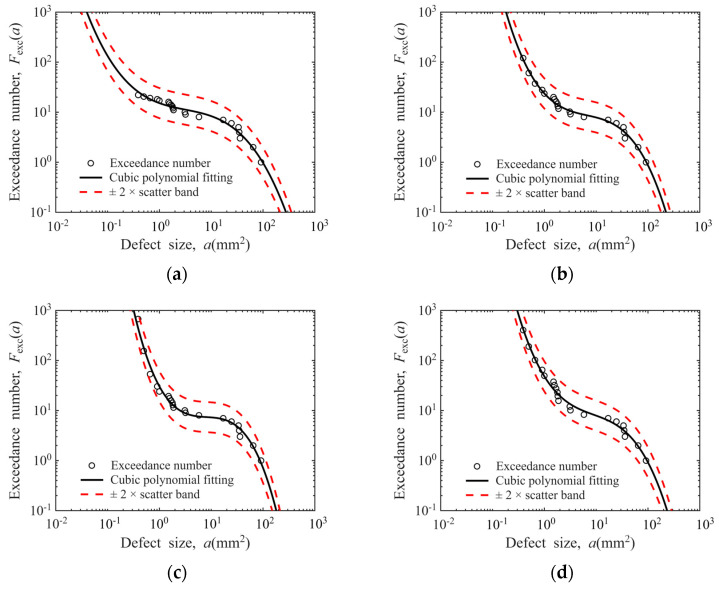
Cubic polynomial model for defect distribution: (**a**) Φ0.8 mm FBH sensitivity in disks; (**b**) Φ1.2 mm FBH sensitivity in disks; (**c**) Φ0.8 mm FBH sensitivity in bars; (**d**) Φ1.2 mm FBH sensitivity in bars.

To establish a more accurate model, cubic polynomial fitting is adopted to estimate the distribution characteristics of defect exceedance numbers, as expressed in Equation (10). The defect distribution curves fitted by this method are plotted in Figure 16, where all the estimated defect exceedance numbers fall within the ±2× scatter band of the model. The model parameters of the regression equations are tabulated in Table 2, with the *R*^2^ values of all four fitted curves verified to be greater than 0.97. These results demonstrate that the cubic polynomial can effectively characterize the distribution characteristics of defects. A detailed discussion on the robustness of the cubic polynomial model is provided in Appendix D.

(10) $$\log(F_{exc}(a))=A\cdot \log^{3}(a)+B\cdot \log^{2}(a)+C\cdot \log(a)+D$$

### 3.4. Defect Distribution Model for Post-Inspection Disks

**Figure 17:**
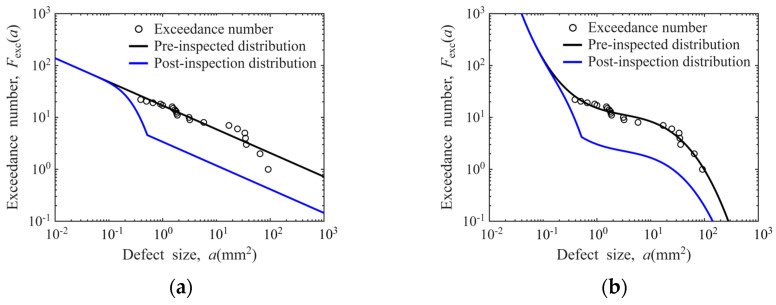
Post-inspection defect distribution model (Φ0.8 mm FBH sensitivity in disks): (**a**) pre-inspection data fitted with linear model; (**b**) pre-inspection data fitted with cubic model.

The post-inspection defect distribution model is derived by integrating the baseline defect distribution of hard-alpha inclusions obtained in Section 3.3 with the POD model established in Section 3.1. During the implementation of the POD model, an upper truncation is imposed on the model to reflect a conservative assumption aligned with practical engineering experience, with the maximum POD value limited to 80% rather than 100% [13]. The post-inspection defect distribution is given by

(11) $$F_{exc\_post}(a)\;\;=\;F_{exc}(a)\;\cdot \left\{1-min[POD(a),0.8]\right\}$$

where the function min[ , ] denotes selecting the minimum value between two arguments. For Φ0.8 mm FBH sensitivity in disks, the result is shown in Figure 17, with curves for other conditions in Appendix E.

### 3.5. Comparison with the Reference Model in AC33.70-3

**Figure 18:**
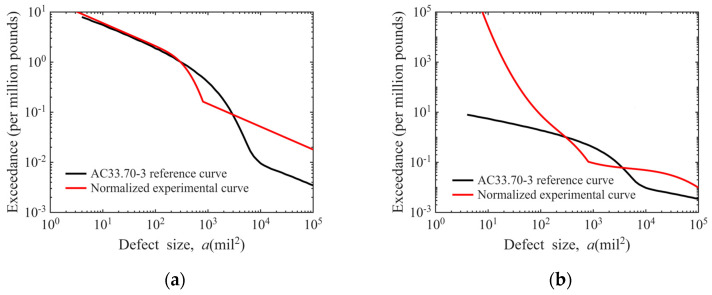
Comparison with reference defect distribution curves in AC 33.70-3: (**a**) linear regression model; (**b**) cubic polynomial model.

The defect distribution curve for “Φ0.8 mm FBH sensitivity in disks” calculated in this work is compared with the “#2 FBH (2/64 inch ≈ 0.7938 mm) Conventional Forging Inspection” curve specified in Advisory Circular AC 33.70-3 issued by the FAA. To enable this comparative analysis, the proposed model undergoes two processing steps: (1) normalize the ordinate defect exceedance number against U.S. field empirical data of one defect per 1,000,000 pounds, with the number of defects at 300 mil^2^ set to one; (2) unify the abscissa defect area unit to mil^2^ (1 mil = 0.0254 mm), the imperial unit used in AC 33.70-3. The resulting comparison is illustrated in Figure 18.

As shown in Figure 18a, linear fitting of the estimated defect exceedance number yields a normalized defect distribution curve with nearly identical trends to the reference curve in AC 33.70-3. For defect sizes below 300 mil^2^ (equivalent to a diameter of approximately 0.5 mm), the two curves almost overlap. For sizes above 300 mil^2^, both curves exhibit a trend of rapid initial decline followed by linear decrease, with defect counts roughly of the same order of magnitude.

As shown in Figure 18b, cubic polynomial fitting of the estimated defect exceedance number results in a new model that exhibits distinct defect distribution trends from the conventional model in AC 33.70-3. The resulting defect distribution curve shows defect counts of the same order of magnitude as the reference curve for sizes above 300 mil^2^. For defects smaller than 300 mil^2^, the defect exceedance number of the new curve grows at a cubic order of magnitude on a logarithmic scale, which is significantly higher than that of the reference curve.

### 3.6. The Impact of Defect Dimensions Estimated by Regression

In the process of POD model construction in Section 3.1, precise CT dimensional measurements were performed for 10 out of the 21 defects, while the dimensions of the remaining 11 defects were extrapolated from the C-scan image data. This section quantifies the impact of this processing method on the model, namely investigating the variability introduced by the linear regression process to both the POD model and the defect distribution model.

**Figure 19:**
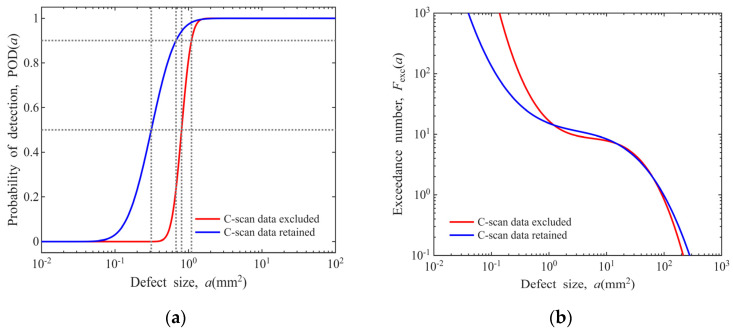
Impact of retaining vs. excluding C-scan estimated defect dimension data (Φ0.8 mm FBH sensitivity in disks): (**a**) POD model; (**b**) cubic polynomial defect distribution model.

A comparative analysis is conducted via data screening, where only the actual CT dimension data of 10 defects are adopted for modeling, with the 11 data points extrapolated from C-scan images excluded. The results are compared with those of the model incorporating all 21 sets of defect data, as presented in Figure 19. As can be seen from the figure, retaining the C-scan estimated data causes the POD curve to shift leftward, and the corresponding defect distribution model yields a lower count of small-sized defects.

**Figure 20:**
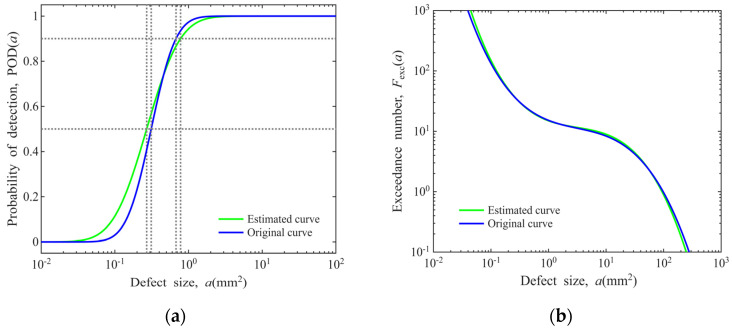
Impacts of linear regression errors on the POD and defect distribution models (Φ0.8 mm FBH sensitivity in disks): (**a**) POD model; (**b**) cubic polynomial defect distribution model.

An uncertainty analysis of the linear regression is also conducted to account for the effects of estimation errors. This issue is illustrated by substituting all 10 sets of actual CT dimension data with the linear regression estimates, with the results presented in Figure 20. The mean errors (*MEs*), variances (*S*^2^), relative mean errors (*RMEs*) and coefficients of variation (*CVs*) for each model are summarized in Table 3. It can be seen that the errors induced by the linear regression estimates of defect dimensions exert an acceptable influence on the POD model and the defect distribution model.

## 4. Conclusions

An experimental method for fabricating disks with porous hard-alpha inclusion defects is proposed using titanium nitride sponge. On this basis, ultrasonic testing experiments are performed on the defect-containing disks and sampling bars, by which a POD model and a defect distribution model specific to porous hard-alpha inclusions are established. Within the scope of the present experimental conditions and sample size, the main findings are summarized as follows:

(1) Porous titanium nitride sponge preforms are introduced during the electrode preparation stage of the smelting process. Through three passes of VAR and forging, TC4 disks with a dimension of Φ625 × 310 mm containing porous hard-alpha inclusion defects are obtained. Unlike the synthetic dense hard-alpha particles prepared via the HIP route, the hard-alpha inclusions fabricated by the method proposed in this work exhibit a porous structure, whose geometric morphology and chemical composition are closer to those of naturally occurring hard-alpha inclusions.
(2) UT experiments are conducted on the hard-alpha inclusions in both the disks and the sampling bars, and POD models are established. For POD modeling, a novel method using high-energy industrial CT scanning is adopted to obtain the actual defect sizes, thus avoiding the destructive dissection of defect-containing specimens. The disk-specific POD models established in this work provide preliminary experimental data for the research on POD models of hard-alpha inclusions in large-sized disks, which has been less reported in existing studies for such specimen specifications.
(3) A modeling approach for deriving the defect distribution model of porous hard-alpha inclusions from the established POD model is explored for the present experimental data, and the derived model is compared with the defect distribution model specified in the current Advisory Circular AC33.70-3. A defect distribution model following a cubic polynomial relationship under logarithmic coordinates is established for the porous hard-alpha inclusions in the tested disks. For the defect data obtained in this study, the log-linear defect distribution model in AC33.70-3 is found to have limited applicability for characterizing the porous hard-alpha inclusion defects in the tested TC4 disks, while the proposed cubic polynomial-based defect distribution model shows a more reasonable fitting effect for estimating the number of small-sized defects (diameter < 0.5 mm) in the tested samples.
(4) Since this study is based on 21 porous hard-alpha inclusions in specific disk specimens, the dataset has limitations. Thus, the cubic polynomial model established herein is currently specifically applicable to the porous hard-alpha inclusions of the studied titanium alloy disks.
(5) Robustness verification shows the model adapts well to ±5% data perturbation and remains robust under ±10% but reaches its robustness boundary at ±20% data perturbation. Therefore, when referring to and using this model under the same test conditions, attention should be paid to this robustness boundary.
(6) The assumptions in defect distribution modeling introduce uncertainty into the model. First, the linear relationship between the C-scan size and maximum CT cross-sectional size of defects: data show the fitted linear relationship causes an RME change of 6.367% and a CV change of 7.157% for the model, both within the acceptable range. Second, the assumed distribution of detected defects: different assumptions lead to differences in the final defect distribution. Using a uniform distribution in this study is feasible due to the lack of field data. If sufficient field data becomes available in the future, a more appropriate assumed distribution could be selected based on actual conditions.

## Figures and Tables

**Table 1:** Parameters of log-log linear regression model for defect distribution.

Test Object	Probe Frequency	Detection Sensitivity	*m*	*n*	*R* ^2^	Outliers
Hard-alpha in disks	10 MHz	Φ0.8 mm FBH	−0.4566	1.2280	0.9043	Retain
			−0.4061	1.2162	0.9352	Exclude
		Φ1.2 mm FBH	−0.6200	1.4362	0.8958	Retain
			−0.5866	1.4284	0.8795	Exclude
Hard-alpha in bars	5 MHz	Φ0.8 mm FBH	−0.7356	1.5694	0.7729	Retain
			−0.7164	1.5649	0.7308	Exclude
		Φ1.2 mm FBH	−0.8304	1.7362	0.8917	Retain
			−0.8142	1.7324	0.8698	Exclude

**Table 2:** Parameters of cubic polynomial model for defect distribution.

Test Object	Probe Frequency	Detection Sensitivity	*A*	*B*	*C*	*D*	*R* ^2^
Hard-alpha in disks	10 MHz	Φ0.8 mm FBH	−0.2265	0.3417	−0.3749	1.1813	0.9711
		Φ1.2 mm FBH	−0.4476	1.1042	−1.1364	1.3743	0.9878
Hard-alpha in bars	5 MHz	Φ0.8 mm FBH	−0.7334	2.0054	−1.8912	1.4818	0.9827
		Φ1.2 mm FBH	−0.4817	1.3696	−1.6721	1.6824	0.9868

**Table 3:** Impacts of linear regression errors on the POD and defect distribution models.

Model Type	*ME*	*S* ^2^	*RME*	*CV*
Linear regression	2.9790	21.4411	9.478%	14.72%
POD model	0.0095	1.32 × 10^−4^	0.986%	1.196%
Defect distribution model	0.7066	0.6309	6.367%	7.157%

**Figure A1:**
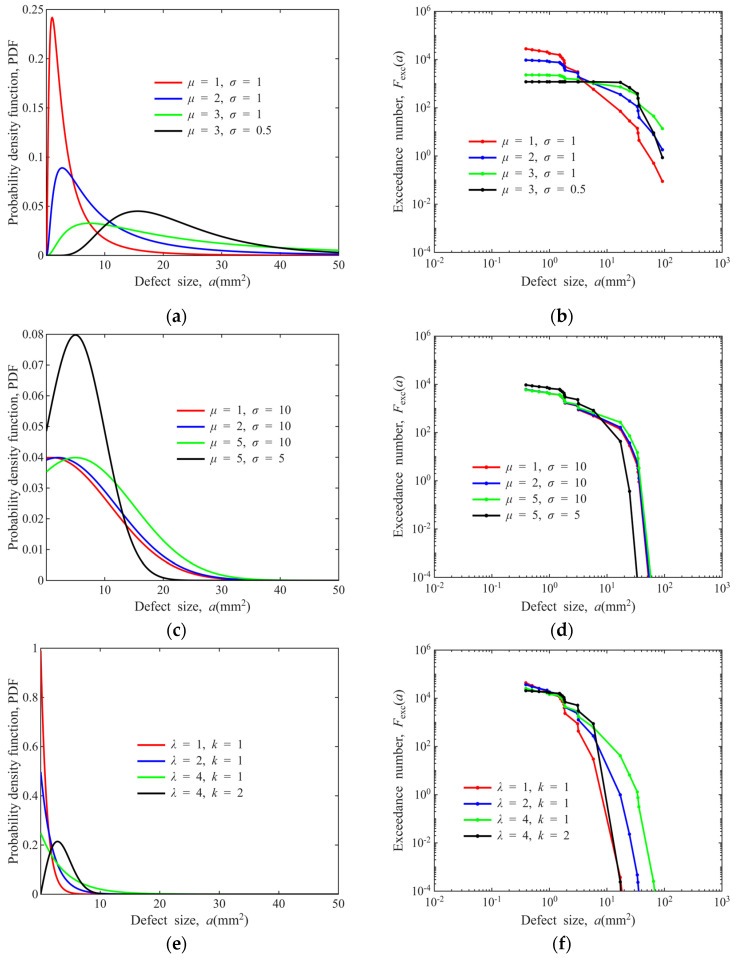
Impact of distributional assumptions of field data on defect distribution models (Φ0.8 mm FBH sensitivity in disks): (**a**) lognormal distribution of *X*(*a*); (**b**) defect distribution models derived from lognormal *X*(*a*); (**c**) truncated normal distribution of *X*(*a*); (**d**) defect distribution models derived from truncated normal *X*(*a*); (**e**) Weibull distribution of *X*(*a*); (**f**) defect distribution models derived from Weibull distribution *X*(*a*).

**Figure A2:**
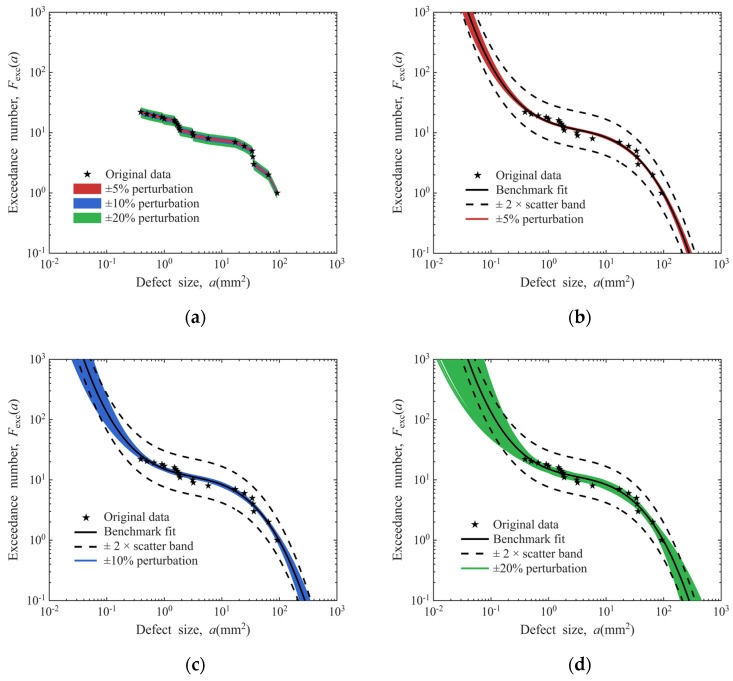
Robustness analysis of cubic polynomial model (Φ0.8 mm FBH sensitivity in disks): (**a**) assumed perturbation of experimental data; (**b**) ±5% perturbation fit envelope; (**c**) ±10% perturbation fit envelope; (**d**) ±20% perturbation fit envelope.

**Figure A3:**
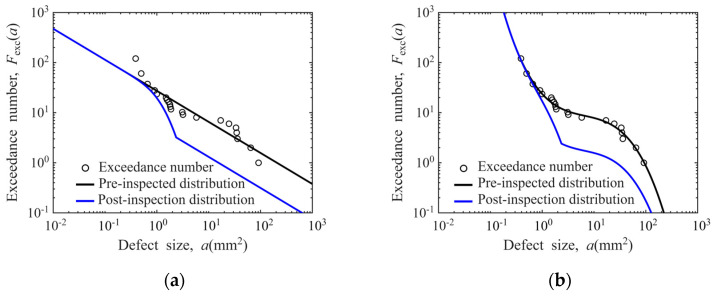

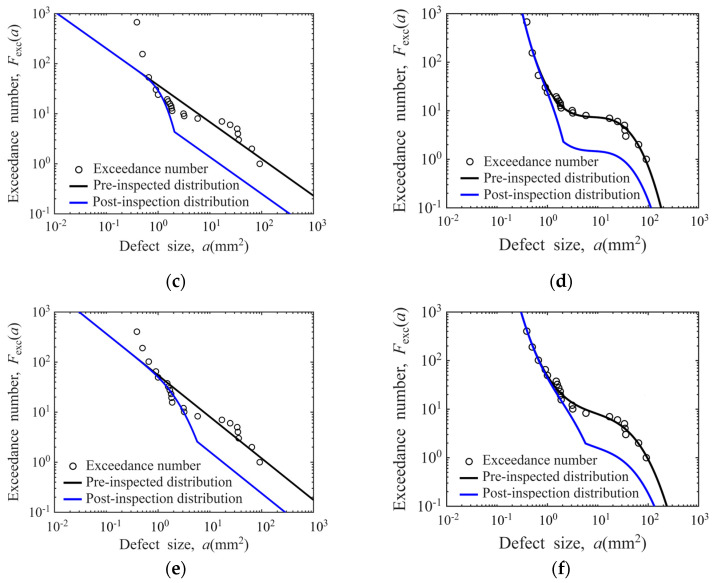
Post-inspection defect distribution models under different detection conditions: (**a**) linear model for Φ1.2 mm FBH in disks; (**b**) cubic model for Φ1.2 mm FBH in disks; (**c**) linear model for Φ0.8 mm FBH in bars; (**d**) cubic model for Φ0.8 mm FBH in bars; (**e**) linear model for Φ1.2 mm FBH in bars; (**f**) cubic model for Φ1.2 mm FBH in bars.

## Data Availability

The original contributions presented in this study are included in the article. Further inquiries can be directed to the corresponding author.

## Appendix A

The three-dimensional morphologies of defects obtained via high-energy CT scanning are presented in Table A1.

**Table A1:** Three-view drawings of defect morphologies.

No.	Front View	Top View	Left View
1			
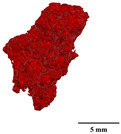
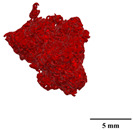
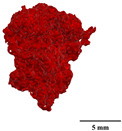
2			
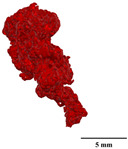
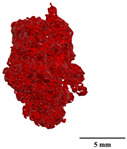
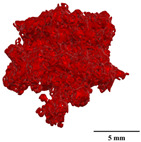
3			
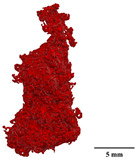
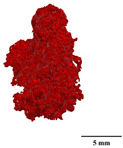
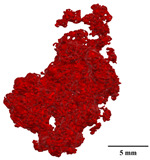
4			
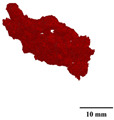
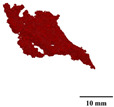
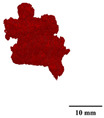
5			
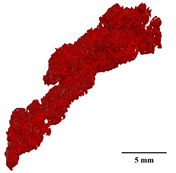
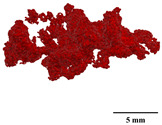
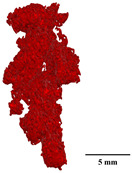
6			
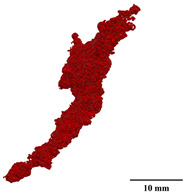
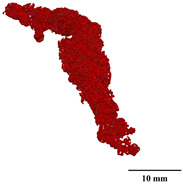
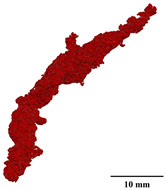
7			
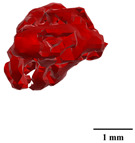
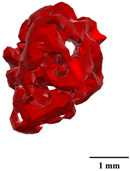
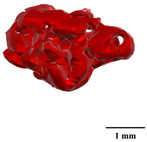
8			
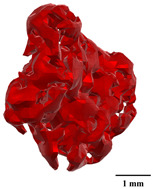
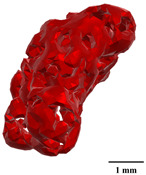
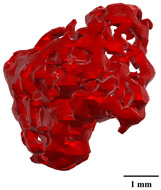
9			
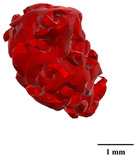
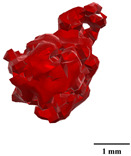
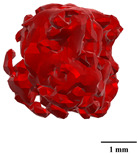
10			
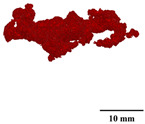
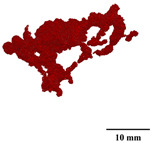
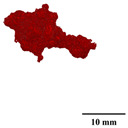

## Appendix B

The actual defect sizes *a* and defect response signals *â* used for POD modeling are presented in Table A2.

**Table A2:** Input data for POD modeling.

No.	Actual Defect Sizes *a* (mm^2^)	Defect Response Signals *â* (mm^2^)		
		Sensitivity for Φ0.8 and Φ1.2 mm FBH in Disks	Sensitivity for Φ0.8 mm FBH in Bars	Sensitivity for Φ1.2 mm FBH in Bars
1	24.62	1.27	0.76	1.63
2	33.69	1.81	0.63	1.63
3	34.61	1.79	1.10	1.49
4	64.46	3.46	1.23	1.75
5	17.07	0.93	0.44	0.86
6	1.79	0.18	0.21	0.22
7	35.95	1.27	0.89	1.39
8	3.19	0.28	0.11	0.14
9	1.82	0.20	0.10	0.06
10	5.80	0.50	0.16	0.26
11	1.68	0.18	0.12	0.11
12	0.39	0.16	0.06	0.12
13	1.87	0.28	0.17	0.29
14	1.50	0.35	0.16	0.13
15	3.10	0.40	0.23	0.37
16	91.81	3.17	1.55	2.26
17	0.50	0.22	0.08	0.12
18	1.00	0.28	0.11	0.28
19	0.91	0.32	0.10	0.14
20	0.66	0.35	0.11	0.16
21	1.57	0.35	0.14	0.31

It should be noted that for the POD modeling of disks, the same defect response signal *â* data are adopted under two different sensitivities. This is because inspectors comprehensively determine the measurement results under the two sensitivities and only retain one set of results as valid data for recording. Nevertheless, the threshold values *â*_dec_ adopted for POD calculations under the two different sensitivities are different (substituted into Equation (A1): *â*_dec_0.8_ = 0.126 mm^2^; *â*_dec_1.2_ = 0.283 mm^2^). Therefore, according to the POD calculation Equation (4) in Section 3.1, two distinct sets of POD curves, as shown in Figure 10a,b, can still be derived.

(A1) $$\left\{\begin{array}{l} \Delta \mathrm{dB}=20\cdot \mathrm{lg}\frac{S_{dec}}{S_{C}}=40\cdot \mathrm{lg}\frac{D_{dec}}{D_{C}}\\ {\hat{a}}_{dec}=\frac{\pi D_{\mathrm{dec}}^{2}}{4} \end{array}\right.$$

where ΔdB represents the decibel difference in the detection signal, $S_{dec}$ denotes the threshold signal value set at 20% FSH, $S_{C}$ denotes the calibrated signal value set at 80% FSH, $D_{dec}$ is the threshold equivalent diameter, and $D_{C}$ is the equivalent diameter of the calibrated FBH.

## Appendix C

In Section 3.2, the distribution function of the detected defects *X*(*a*) is assumed to follow a uniform distribution due to the complete absence of field data. Here, to examine the sensitivity of the results to different assumed distributions, the lognormal distribution, truncated normal distribution, and Weibull distribution are employed to represent the distribution of *X*(*a*), respectively, and the defect distributions obtained from the corresponding calculations are presented in Figure A1.

It can be seen from Figure A1 that different assumed distributions *X*(*a*) yield distinct effects. Based on the calculation results in the figure, some preliminary qualitative insights can be obtained: (1) for the lognormal distribution, the defect distribution transitions from steep to smooth with an increase in the model parameter *μ*; (2) for the truncated normal distribution, the model parameter *σ* has a more significant effect than *μ*; (3) for the Weibull distribution, variations in the model parameters exert a notable influence on the estimated number of large-sized defects; (4) different assumed distributions *X*(*a*) lead to significantly different estimated values of the defect counts and curve shapes.

In contrast to the parameters of other assumed distributions *X*(*a*) that exert a significant influence on the final results, the uniform distribution assumption circumvents this issue. Arguably, it represents a simple and feasible option in the complete absence of field data.

## Appendix D

In Section 3.3, a cubic polynomial model for defect distribution is proposed, and the defect distribution curves corresponding to four different test conditions are derived. This preliminarily validates the good generalization ability of the model. In this appendix, the robustness of the proposed model is further investigated and discussed.

The detailed procedure is as follows: First, three perturbation levels of ±5%, ±10% and ±20% are defined based on the original defect distribution data obtained from experiments, and three corresponding sample spaces are generated accordingly. Second, for each sample space, 1000 sets of perturbed defect distribution exceedance numbers are generated. A new cubic polynomial curve is fitted to each set of the perturbed exceedance numbers, resulting in three groups of curves with 1000 curves per group. Third, the envelope spaces formed by these perturbed exceedance number curves are compared with the benchmark fit derived from the original unperturbed data. The results are presented in Figure A2.

Figure A2 shows that the cubic polynomial model can adapt well to the variations caused by perturbations in the experimental data, exhibiting favorable robustness. Specifically, under the ±5% perturbation, all cubic polynomial curves corresponding to the 1000 new samples fall within the ±2× dispersion band of the benchmark fit. Under the ±10% perturbation, the 1000 new curves mostly lie within the ±2× dispersion band, with only a small portion exceeding it when the defect size is less than approximately 0.1 mm^2^. For the ±20% perturbation, the new curves still demonstrate good adaptability within the defect size range with actual experimental values yet deviate significantly from the ±2× dispersion band of the benchmark fit when the defect size is less than about 0.2 mm^2^ or greater than about 100 mm^2^.

On this basis, three statistical parameters are further employed to quantitatively analyze the effects of perturbations on the model, namely the coefficient of determination (*R*^2^), sum of squared errors (*SSE*), and root mean square error (*RMSE*). The results are presented in Table A3. It can be seen that data perturbations have a negligible effect on *R*^2^. For *SSE* and *RMSE*, the model exhibits good robustness against minor perturbations (±5%) with only slight fluctuations in fitting deviations. It remains adaptable to moderate perturbations (±10%). When the perturbation amplitude reaches ±20%, SSE and RMSE change significantly, indicating that the model has reached its critical robustness boundary.

**Table A3:** Statistical parameter variations under different perturbation levels.

Perturbation Level	*R*^2^ (Range, Δmax%)	*SSE* (Range, Δmax%)	*RMSE* (Range, Δmax%)
Original data (0%)	0.9711, —	0.0738, —	0.0593, —
±5%	[0.9619, 0.9781], 0.95%	[0.0559, 0.0972], 31.7%	[0.0516, 0.0680], 14.7%
±10%	[0.9496, 0.9827], 2.14%	[0.0441, 0.1285], 74.0%	[0.0458, 0.0782], 31.9%
±20%	[0.9153, 0.9847], 5.57%	[0.0391, 0.2160], 192.6%	[0.0431, 0.1014], 71.0%

## Appendix E

The post-inspection defect distributions under different conditions are presented in Figure A3.
